# Determination of aromatic amines in human urine using comprehensive multi-dimensional gas chromatography mass spectrometry (GCxGC-qMS)

**DOI:** 10.1007/s00216-014-8080-5

**Published:** 2014-08-21

**Authors:** Xolelwa Lamani, Simeon Horst, Thomas Zimmermann, Torsten C. Schmidt

**Affiliations:** 1 Instrumental Analytical Chemistry, University of Duisburg-Essen, Universitätstrasse 5, 45141 Essen, Germany; 2 Actelion Pharmaceuticals Ltd, Gewerbestrasse 16, 4123 Allschwil, Switzerland; 3 Centre for Water and Environmental Research ZWU, University of Duisburg-Essen, Universitätsstrasse 2, 45141 Essen, Germany

**Keywords:** Aromatic amines, Human urine, Bladder cancer, Comprehensive multidimensional gas chromatography, Solid phase micro-extraction (SPME), Ionic liquid columns

## Abstract

Aromatic amines are an important class of harmful components of cigarette smoke. Nevertheless, only few of them have been reported to occur in urine, which raises questions on the fate of these compounds in the human body. Here we report on the results of a new analytical method, in situ derivatization solid phase microextraction (SPME) multi-dimensional gas chromatography mass spectrometry (GCxGC-qMS), that allows for a comprehensive fingerprint analysis of the substance class in complex matrices. Due to the high polarity of amino compounds, the complex urine matrix and prevalence of conjugated anilines, pretreatment steps such as acidic hydrolysis, liquid–liquid extraction (LLE), and derivatization of amines to their corresponding aromatic iodine compounds are necessary. Prior to detection, the derivatives were enriched by headspace SPME with the extraction efficiency of the SPME fiber ranging between 65 % and 85 %. The measurements were carried out in full scan mode with conservatively estimated limits of detection (LOD) in the range of several ng/L and relative standard deviation (RSD) less than 20 %. More than 150 aromatic amines have been identified in the urine of a smoking person, including alkylated and halogenated amines as well as substituted naphthylamines. Also in the urine of a non-smoker, a number of aromatic amines have been identified, which suggests that the detection of biomarkers in urine samples using a more comprehensive analysis as detailed in this report may be essential to complement the approach of the use of classic biomarkers.

**Graphical Abstract:**
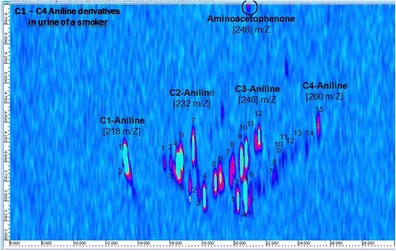
ᅟ

ᅟ

## Introduction

Aromatic amines are highly polar organic compounds that are used or produced in various fields such as drugs, pesticides, dyes, tobacco smoke, and intermediates for the manufacturing of other chemicals [1, 2]. Aromatic amines in tobacco smoke are suggested to be important causing agents for the development of bladder cancer in humans [3]. In early studies, human bladder cancer was frequently associated with occupational exposure to carcinogenic aromatic amines such as 4-aminobiphenyl, 2-napthylamine, 2-methylaniline, and 4,4′-diaminobiphenyl as well as to some polycyclic aromatic hydrocarbons (PAHs), and since it has been known that these chemicals are carcinogenic to humans, their exposure was highly reduced [2, 4]. However, in the 1950s the composition of tobacco and cigarette design changed, resulting in high concentrations of bladder carcinogens such as 2-naphthylamine to occur in cigarette smoke, and hence an increased risk of smoking-related bladder cancer [5, 6]. The International Agency for Research on Cancer (IARC) so far classified six aromatic amines as carcinogenic (4-aminobiphenyl, 2-naphthylamine, and 4,4′-diaminobiphenyl) or probably carcinogenic [4-chloro-2-methylaniline, 2-methylaniline, 4,4′-methylene bis (2-chloraniline)] to humans [7]. They suggested tobacco smoking to be a major cause of transitional-cell carcinoma (TCC), the most common type of bladder cancer, where the risk increases with the duration of smoking and the number of cigarettes smoked [4, 8, 9]. A cigarette is known to contain up to 600 ingredients and, when burned, it produces more than 4000 chemicals, of which some are known to be carcinogenic. Among these, some aromatic amines are found to be present both in the main stream and in the side stream smoke [3, 10]. During the smoking process, aromatic amines are carried into the bloodstream where they are filtered out of the blood by the kidneys and end up in the urine. When the urine is stored in the bladder, aromatic amines get in contact with the cells that line the bladder where they react and may form high-grade cancer cells [4]. There is still limited information about the amount and/or specific aromatic amines that can cause bladder cancer in humans. There is evidence, though, that more anilines have to be considered as several amines were mutagenic in toxicologic testing or caused cancer in testing animals [11], but the results are difficult to extrapolate to humans. In the past few years, several analytical methods have been published on the analysis of aromatic amines in human urine [3, 12–14]. However, the studies were somehow limited to analysis with conventional gas chromatography, where many isomeric compounds may not be separated due to their similar retention times and, as a result, a rather small number of aromatic amines are detected in urine. To our knowledge, no results have yet been reported where analysis of aromatic amines in urine was made by comprehensive two-dimensional chromatography. The method described here offers a combination of very sensitive and precise analytical techniques that do not only isolate and extract the analytes from the matrix but also decrease their polarity and introduce iodine to the aromatic ring for the analytes to be detected selectively. Furthermore multi-dimensional gas chromatography mass spectrometry (GCxGC-qMS) in combination with solid phase microextraction (SPME) was applied to enrich, separate, and make it possible to distinguish isomers of several aromatic amines of the same molecular mass. In addition to the method development, ionic liquid columns (ILC) were tested to validate if their use could possibly offer better selectivity for iodinated derivatives. The columns covered polar, highly polar, and extremely polar stationary phases.

This study demonstrates exemplarily patterns of aromatic amines found in urine of a smoker and non-smoker due to their potent carcinogenic effect that can be caused by tobacco smoking and may lead to bladder cancer.

## Experimental

### Chemicals and reagents

Aromatic amines and reference substances with a purity of >97 % were purchased from Sigma-Aldrich, Steinheim, Germany and Alfa-Aesar, Karlsruhe, Germany. The reagents used for hydrolysis and derivatization, namely concentrated hydrochloric acid (37 %), hydriodic acid (55 %, unstabilized, A.C.S.), sodium nitrite (≥97 %), sodium sulphite (≥98 %), alizarinsulfonic acid (98 %), and amidosulfonic acid (99, 3 %) were all products of Sigma-Aldrich. Sodium hydroxide (99 %) was from VWR, Darmstadt, Germany, and sodium acetate (≥99 %) from Applichem, Darmstadt, Germany. Analytical grade methanol was purchased from KMF Laborchemie, Lohmar, Germany, and the ultra-filtered water used was a product of PureLab Ultra (ELGA LabWater, Celle, Germany).

### Stock solution and standard preparation

Stock solutions were prepared by weighing approximately 10 mg of each pure substance in a 10-mL volumetric flask and diluting it with analytical grade methanol to make a final concentration of 1 g/L. From the stock solution, standard solutions with concentrations from 1 ng/L to 500 ng/L were prepared in an aqueous medium by dilution using Hamilton syringes. The stock solution, as long as it was not used, was kept cool in a refrigerator at 4 °C for at most 1 mo. The diluted standards were freshly prepared from the stock solution for every analysis.

### Urine sample preparation

Urine samples were collected randomly from smoking and nonsmoking donors in 50-mL polypropylene tubes. Into 20 mL urine sample, 10 mL concentrated hydrochloric acid (37 %) was added and the mixture was heated in properly closed vials for 12 h at 110 °C to cleave aromatic amine adducts. After cooling to room temperature, the samples were made alkaline by adding 20 mL of a 10-*M* sodium hydroxide solution so that the amines will be present in their non-charged form. During this process, a black precipitate forms that could disturb extraction and, therefore, the samples had to be filtered using a filter paper. The filtered samples were extracted three times, with 5-mL diethylether while shaken manually for about 1 min and then left to stand for another 1 min to allow the separation of aqueous and organic layers. The aqueous phase was discarded and the organic phase was kept and washed once with 2 mL of 0.1 *M* sodium hydroxide solution. The amines in the organic phase were extracted back into the aqueous phase with 5 mL water acidified with 100 μL concentrated hydrochloric acid (37 %). The remaining diethylether in the aqueous samples was gently evaporated in a nitrogen stream for 5 min. Urine samples of both smoker and non-smoker were prepared in duplicate.

### Derivatization

To decrease the polarity of the extracted amines as well as introducing iodine in the aromatic ring, the samples were derivatized through diazotization and iodination in a one-pot reaction, as partly described by Schmidt et al. [15]. Into each 5 mL extracted sample, 100 μL hydriodic acid (55 %) and 200 μL sodium nitrite (50 g/L) were added and the sample was shaken for 20 min. During the reaction, the aromatic amines are diazotized followed by a subsequent substitution by iodine at the aromatic ring, which results in aromatic iodine compounds. To destroy the surplus of nitrite, 0.5 mL of amidosulfonic acid (50 g/L) was added. The sample was shaken for another 45 min and then heated in a water bath at 95 °C for 5 min to convert the unreacted diazonium ions to phenols and also to decompose excess amidosulfonic acid. After cooling to room temperature, 125 μL saturated sodium sulfite was added to reduce the iodine residue. This is demonstrated by immediate discoloration of an initially brownish solution. Finally, a 100 μL of 1 alizarinsulfonic acid was added to the sample followed by 0.5 mL of saturated sodium acetate to adjust the pH of the sample to pH 5.

### SPME

Headspace SPME was used to enrich the iodinated derivatives before measuring by GCxGC-qMS. Prior to extraction, the headspace vial containing the sample was pre-incubated for 10 s at the incubation temperature of 60 °C, while agitating at the speed of 500 rpm. The SPME fiber was then auto-injected into the headspace vial to extract the solution in the headspace for 25 min, followed by desorbing the extracted analytes into the GC-injection port for 5 min. After every extraction, the SPME fiber was conditioned in the needle heater for 20 min. For the analyses of authentic standards and urine samples, the same extraction conditions with the SPME fiber were used and the solutions were extracted and measured once. To determine the extraction efficiency of the SPME fiber, depletion SPME experiments were done by analyzing four reference substances, namely iodobenzene, methyl-iodobenzene, chloro-iodobenzene and pentafluoro-iodobenzene. These substances are the derivatives of very common aromatic amines except for pentafluoro-iodobenzene, which was used as an internal standard. A 5 μg/L mixture of these four compounds was prepared in a 10-mL volumetric flask and made up to the mark with water; 2 mL of the solution was transferred into a 20-mL headspace vial and diluted with 8 mL water to make a final concentration of 1 μg/L, in triplicate. Each solution was extracted with the SPME fiber and measured five times consecutively.

### Instrumentation

All analyses were performed by a Shimadzu GC system consisting of a GC-2010 and GCMS QP2010 Plus (Shimadzu GmbH, Duisburg, Germany) with a built-in loop-type modulator from Zoex Corporation (Houston (Texas), USA). The system is coupled with a Shimadzu AOC-5000 liquid, headspace, and SPME GC injection system, which has a control panel for mobilizing samples and injectors. The SPME fiber used for sample enrichment was 65 μm PDMS/DVB (Sigma-Aldrich, Schnelldorf, Germany) with a SPME liner of 0.75 mm × 5.0 mm × 95 mm made for Shimadzu GCs. The GC columns used were, for the first dimension, a DB-5, 30 m, 0.25 mm i.d., 0.25 μm film from Agilent Technologies, Waldbronn, Germany and for the second dimension, a BPX-50, 2.7 m, 0.15 mm i.d., 0.15 μm film from SGE Analytical Science, Griesheim, Germany. Ionic liquid columns namely SLB IL-59 (polar), SLB IL-61 (polar), SLB IL-76 (highly polar), SLB IL-82 (highly polar), and SLB IL-100 (extremely polar) were supplied by Sigma-Aldrich, Taufkirchen, Germany with 2.7 m, 0.1 mm i.d. and 0.08 μm film. The second column was coiled around the Zoex loop-type modulator allowing 1 meter after cryo-focusing to the detector. The modulation time was set at 6 s. Helium was used as carrier gas and the nitrogen cooled by liquid nitrogen for cryogenic effect. In the case where GC/MS was used, the cold jet was set off, hence no modulation and cryogenic effect was needed. The column temperature gradient was set initially at 60 °C and held for 3 min, then increased to 230 °C at 5 °C/min and held for another 3 min. At this start temperature (60 °C) the column head pressure was 97 kPa. The column flow was set at 1 mL/min with a linear velocity of 29 cm/s. The temperature of injection port and MS interface were set at 250 °C and the ion source temperature at 230 °C. The instrument was operated in the splitless injection mode with the detector voltage at 1.2 kV and scan rate of 10,000 u/s in full scan mode. The scanned mass-to-charge ratios were between *m/z* 70 and 300 for authentic standards (scan speed = 0.02) and *m/z* 70–460 for urine samples (scan speed = 0.03). The data was processed via GCxGC Software, GC Image (Shimadzu GmbH, Duisburg, Germany).

### Safety considerations

Some pure substances of aromatic amines are highly toxic and/or carcinogenic. Therefore, care should be taken during the preparation of stock solutions by strictly using protection wear such as nose mask, hand gloves, laboratory coat, etc. as well as working strictly under the fume hood. Some symptoms of contact with the substances are itching skin, irritation in the nose, and sudden sharp headaches when inhaled.

## Results and discussion

### Determination of extraction ratio by depletion SPME

**Fig. 1:**
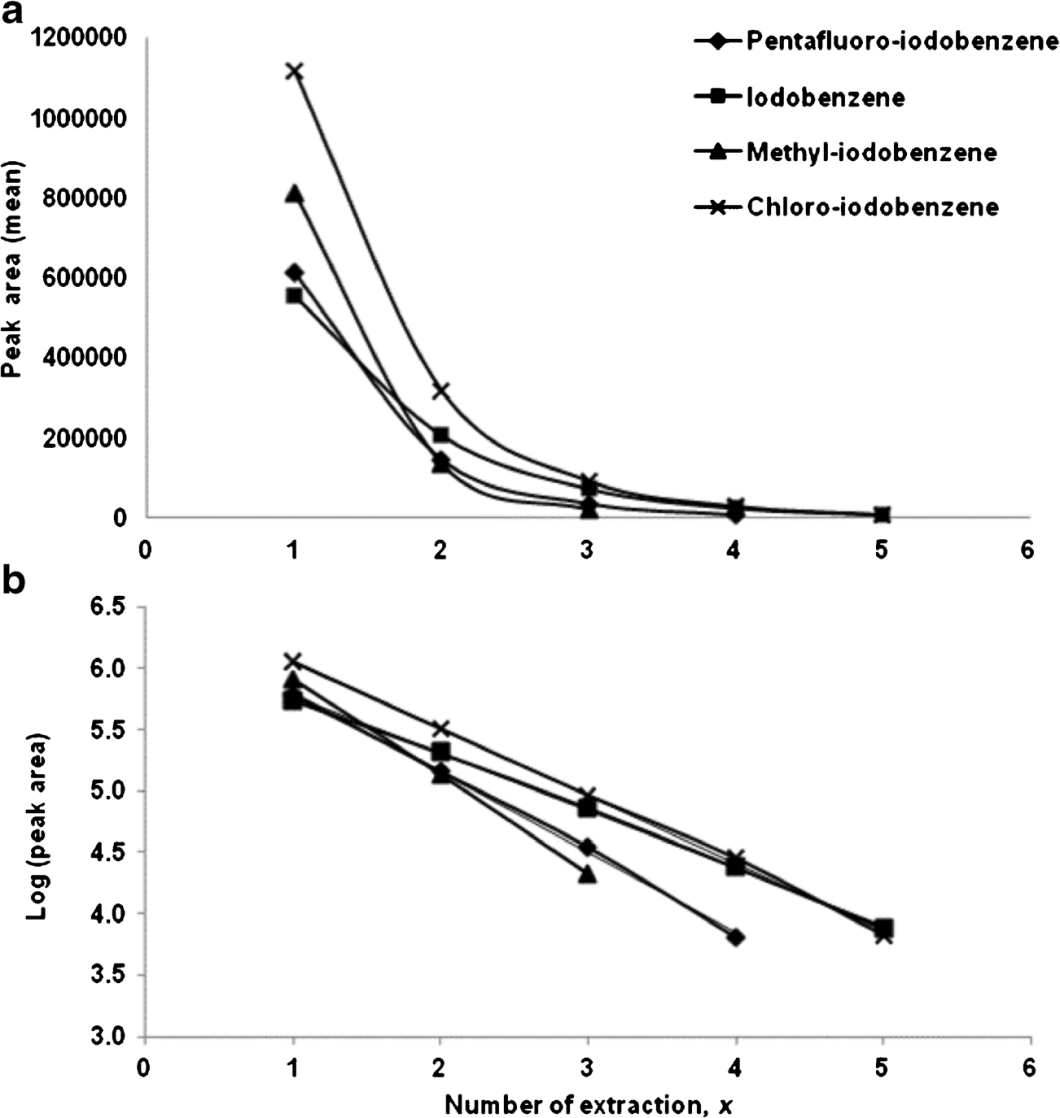
Multiple extraction of a sample containing four iodinated compounds at a concentration of 1 μg/L using a 65 μm PDMS/DVB SPME fiber and the determination of peaks area with GC/MS. In (**a**) mean peak area and in (**b**) log (peak area) are plotted against the number of extraction, *x* where n = 3. Relative standard deviations (RSD) were ≤7 % for the first two extractions and ≤20 % for extractions 3 to 5. The RSDs are not shown here to increase legibility

To test the depletion SPME model as described by Zimmermann et al. [16], a 10 mL sample (n = 3) containing four reference substances at a concentration of 1 μg/L was analyzed. A 65 μm PDMS/DVB fiber was used where each sample placed in a 20-mL headspace vial was extracted in the headspace (vial penetration of 22 mm) and analyzed by GC/MS five times consecutively. The conditions for extraction with the SPME fiber are as described previously. The exponential decay of the peak areas for each analyte, in correlation with the number of extraction, was observed. This is demonstrated in Fig. 1a where the mean peak areas for each analyte are plotted against the number of extraction (*x*). For methyl-iodobenzene, more than 80 % of the analytes were extracted after the third extraction time and no more analytes could be extracted from the solution. Hence, the number of extractions (*x*) shown is up to three. The same applies for pentafluoro-iodobenzene with the number of extraction (*x*) shown up to four. To determine the SPME fiber extraction efficiency, the following equation was used:

1 $$ {n}_{f, x} = {n}_{s, o} E{\left(1\hbox{-} E\right)}^{x\hbox{-} 1} $$

where *n*_*f,x*_ is the extracted amount of analytes, *n*_*s,o*_ the initial amount of analytes in the sample, *E* the extraction ratio, and *x* the number of the consecutive extraction steps. The results can be compared with the fitting function *f(x) = ab* ^*x*^ with *a = n*_*s,o*_ *E* and *b = 1- E*. From this equation, the extraction ratio *E* could be easily determined from the slope *b* after linearization:

2 $$ \log {n}_{f, x}= \log \left({n}_{s, o} E\right)+\left( x-1\right) \log \left(1\hbox{-} E\right) $$

Figure 1b shows the log peak areas plotted against the number of extraction, *x*.

The slopes obtained from the plots and the calculated extraction ratios of the analytes are shown in Table 1 with extraction efficiency of the SPME fiber ranging between 65 % and 85 %, with confidence intervals between 5.8 % and 7.1 % and RSD of 4 %–7 %. The fiber-sample partition coefficient (*K*_*fs*_) of each compound was calculated from the extraction ratios using the equation below:

3 $$ {K}_{f s}={V}_s E/{V}_f\left(1\hbox{-} E\right) $$

where *K*_*fs*_ is the partition coefficient between the fiber coating *f* and the aqueous sample *s* and *V*_*s*_ and *V*_*f*_ are the volumes of the sample (10 mL) and the fiber coating (0.440 μL), respectively. Due to the good precision and the efficient extraction of the sample, the PDMS/DVB fiber was retained for further experiments, and hence no other SPME fibers were tested.

**Table 1:** Calculated extraction ratios, *E* and fiber sample partition coefficients, *K*_*fs*_ for depletion SPME

Compound	R^2^	Slope [log (1-E)]	E (%)	K_fs_
Pentafluoro-iodobenzene	0.9981	–0.6565	78 ± 6.2	80579
Iodobenzene	0.9991	–0.4657	66 ± 5.8	43727
Methyl-iodobenzene	0.9998	–0.7950	84 ± 7.1	119318
Chloro-iodobenzene	0.9989	–0.5489	72 ± 6.0	57581

### Method development for GCxGC using pure substances

**Fig. 2:**
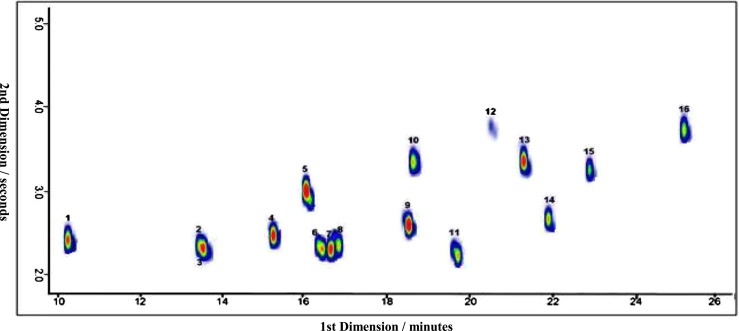
A 100 ng/L standard mixture of 16 derivatized aromatic amines and analysis by GCxGC-qMS for the method development. The analytes are numbered based on their elution time and are listed in Table 2

A standard solution with 16 aromatic amines at 100 ng/L in aqueous solution was used to optimize instrument parameters and to develop a method for the determination of aromatic amines in urine samples. The standards were derivatized as described in the Experimental section and analyzed by GCxGC-qMS. In Fig. 2, a 100 ng/L standard is shown as an example and Table 2 summarizes the names, the derivative fragments (*m/z*), and estimated LODs of the analyzed substances. All the compounds were eluted and well separated within 30 min in the first-dimension (1D) and within 6 s in the second-dimension (2D), except for the methylaniline derivatives (analytes 2 and 3), which could not be separated. Conversely, dimethylaniline derivatives (analytes 7 and 8) were well separated. The mass spectra of all derivatives showed the typical iodine fragment ion signal with 127 *m/z*, which enables the analytes to be detected selectively, even in complex samples such as urine. The method was used further to detect aromatic amines in urine samples.

**Table 2:** Names and fragments of the iodinated derivatives analyzed for method development with estimated LODs indicated for aqueous standards

No.	Substance	Derivatives	Fragments of the iodinated derivatives (*m/z*)	LODs estimated from calibration [17] in ng/L
1	Aniline	Iodobenzene	204, 77	24.4
2	o-Methylaniline	1-Iodo-2-methylbenzene	218, 91	5.2^a^
3	p-Methylaniline	1-Iodo-4-methylbenzene	218, 91	5.2^a^
4	3-Chloro-4-fluoroaniline	3-Chloro-4-fluoro-1-iodobenzene	256, 129	18.8
5	2-Chloroaniline	2-Chloro-1-iodobenzene	238, 111	3.8
6	4-Ethylaniline	4-Ethyl-1-iodobenzene	232, 217,105	11.4
7	2,6-Dimethylaniline	2,6-Dimethyl-1-iodobenzene	232, 105	9.3
8	2,4-Dimethylaniline	2,4-Dimethyl-1-iodobenzene	232, 105	9.3
9	4-Chloro-2-methylaniline	4-Chloro-1-iodo-2-methylbenzene	252, 125	4.3
10	2-Bromoaniline	2-Bromo-1-iodobenzene	282, 155	10.9
11	2,4,6-Trimethylaniline	1-Iodo-2,4,6-trimethylbenzene	246, 119	22.4
12	2-Aminoacetophenone	2-Iodoacetophenone	246, 203, 76	n.a.
13	2,6-Dichloroaniline	2,6-Dichloro-1-iodobenzene	272, 145	6.1
14	3-Chloro-2,6-dimethylaniline	3-Chloro-2,6-dimethyl-1-iodobenzene	266, 139	13.4
15	3-Chloro-4-methoxyaniline	3-Chloro-1-iodo-4-methoxybenzene	268, 141	13.1
16	2-Naphthylamine	2-Iodonaphthalene	254, 127	19.3

n.a.: No calibration performed due to low signal intensity below 100 ng/L.

^a^ Compounds 2 and 3 could not be separated and therefore the sum of both was integrated.

### Choice of second dimension column

**Fig. 3:**
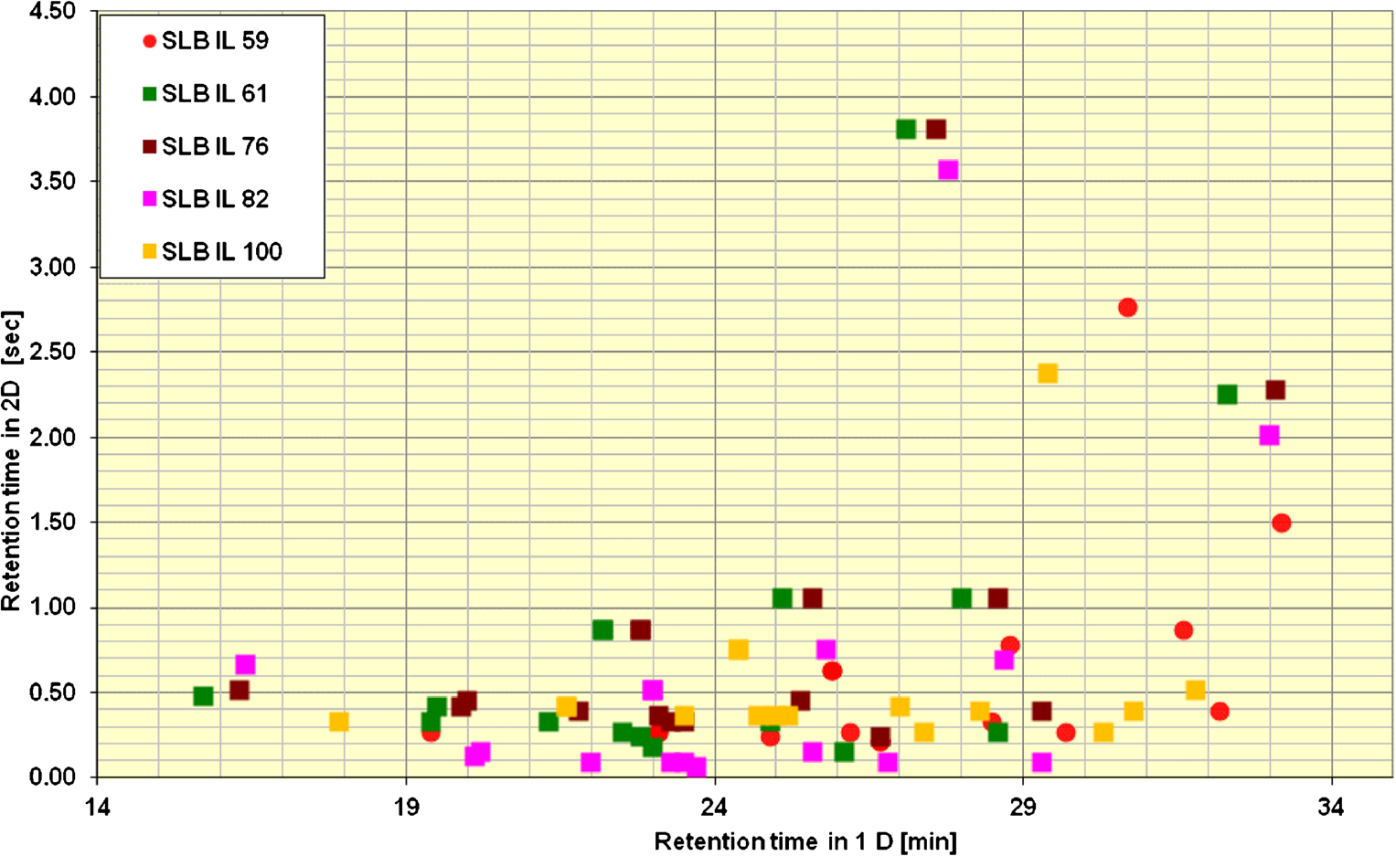
Distribution of the 15 derivatized aromatic amines peaks mentioned in Table 2 (except for 3-chloro-4-methoxyaniline) on the DB-5/ ILC combinations and analysis by GCxGC-qMS

In order to investigate further the separation of isomers in the second dimension, five ILC were studied and compared with the standard BPX-50 column. The columns, in the order of increasing polarity were: SLB IL - 59, 61, 76, 82, and 100. In the first dimension, the same column was kept throughout the experiments (DB-5 column, non-polar). For these experiments, a standard consisting of a mixture of aromatic amines mentioned in Table 2, except for 3-chloro-4-methoxyaniline, was used. The standards were derivatized and enriched by headspace SPME as described before and analyzed by GCxGC-qMS. To compare the column combinations, peak distribution of the analytes in 2D space was plotted for all five ILC as shown in Fig. 3 below.

The analytes were better spread in one of the polar IL columns, IL 61, and two of the highly polar columns, IL 76 and 82. The results obtained imply that different ionic liquids interact in a comparable way with the iodinated derivatives analyzed here as the retention times in the second dimension are similar. Another possibility could be that as compounds elute from the first dimension they are already near the elution temperature for the second column and are, therefore, poorly retained. The results are comparable to the BPX-50 column, a traditional mid-polar GC column used in the experiments mentioned previously in this study (for example Fig. 2). As interesting as the ILC features are, BPX-50 was found to be as well suited for our analytes of interest and, therefore, was retained for further analysis.

### Aromatic amines in urine samples

**Fig. 4:**
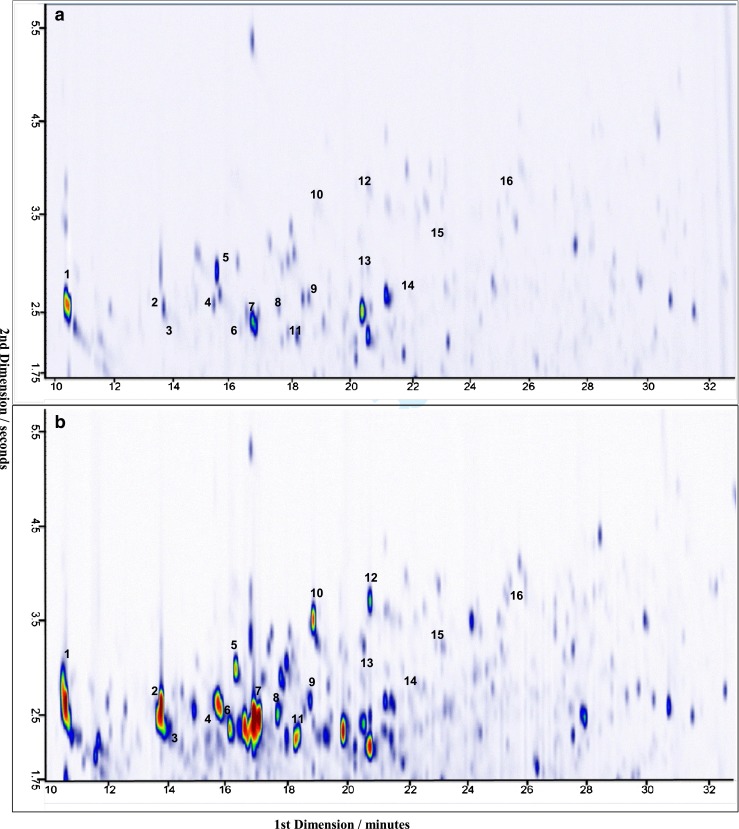
TIC from *m/z* 70–460 showing aromatic iodine compounds proposed to be present in the urine of (**a**) a person without exposure to cigarette smoke and (**b**) a smoking person. The same scale was used on both chromatograms. The red color indicates the high intensity of the analyte followed by yellow, green, and blue as the intensity decreases. The samples were measured by SPME GCxGC-qMS after hydrolysis, liquid–liquid extraction, and derivatization. Numbers in the figure refer to compounds listed in Table 2 and shown in Fig. 2. Note that not all of the shown spots are necessarily caused by aniline derivatives

**Fig. 5:**
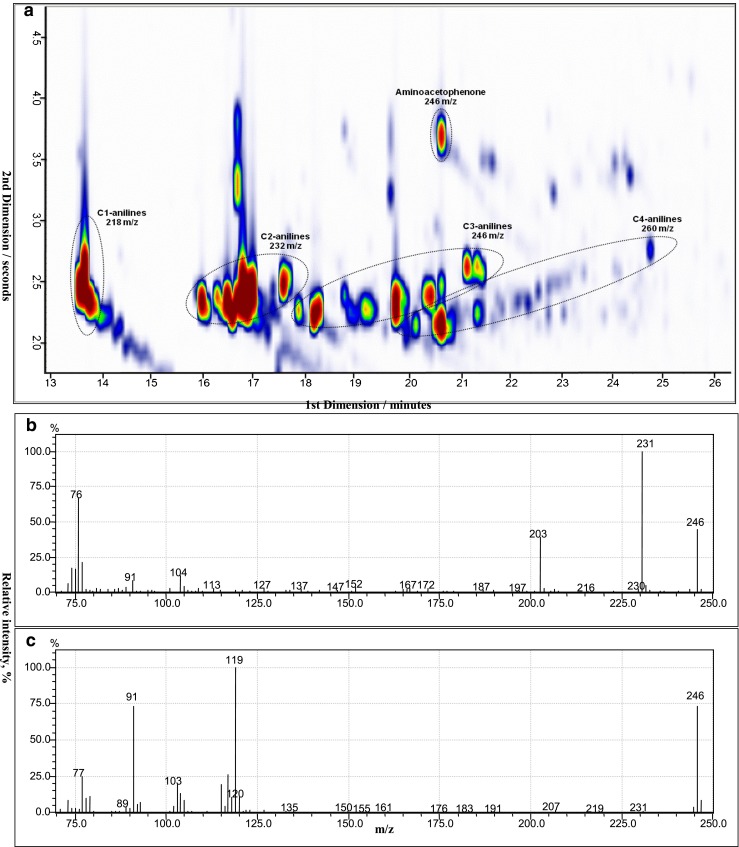
(**a**) Reconstructed ion chromatogram (RIC) of a urine sample of a smoker showing typical *m/z* associated with homologous alkylated aromatic iodine compounds and a separation of aminoacetophenone and C3-aniline derivatives with the same mass-to-charge ratio, *m/z* 246. The compounds selected are derivatives of C1-anilines (*m/z* 218), C2-anilines (*m/z* 232), C3-anilines/aminoacetophenone (*m/z* 246), and C4-anilines (*m/z* 260), and the RIC is generated by summing up signals at these four *m/z*; (**b**) and (**c**) show mass spectra of iodinated derivatives of aminoacetophenone and C3-aniline, respectively, with fragmentation patterns and loss of *m/z* 127 (iodine)

Urine samples of a tobacco smoker and a non-smoker were hydrolyzed, extracted, derivatized, and analyzed by GCxGC-qMS. The analyses were carried out in full-scan mode so that all extractable and GC-suitable compounds were detected. Figure 4a and b show the total ion chromatogram (TIC) of the aromatic iodine compounds that are proposed to be present in the urine of non-smoking and smoking donors, respectively. In both Fig. 4a and b, not all the compounds can be visualized because of low intensity, especially in the urine of a non-smoker, but their identification is possible. The mass spectra of all derivatives showed the typical iodine fragment ion signal with 127 *m/z*; as a consequence, aromatic amines present in the samples could be identified by monitoring the iodine fragment ion. Since intensity of the mass fragment 127 is typically small, it would be beneficial to monitor instead the neutral loss of iodine. However, so far there is no opportunity in GCxGC software to do so. For each compound, the isomers (viz. blobs) were counted to obtain the total sum of analytes present in the sample. This is summarized in Table 3 with the names of the anilines and the fragment ions of the iodinated derivatives. In the urine of a donor exposed to cigarette smoke, ca. 150 aromatic amines were tentatively identified. Among the substances found, less than 10 are described in literature in relation with tobacco smoking–bladder cancer [2, 4, 8]. In the urine of a person without known exposure to cigarette smoke, many anilines (ca. 120) were still identified; nonetheless, regarding the number and peak intensities of occurring iodinated derivatives, the sample was less burdened. Amongst the detected compounds, alkylated anilines were the largest group (n = 39), as shown in Fig. 5a in groups of isomeric homologues (C1- to C4-anilines). Here, the number of isomers rapidly increases with larger or more numerous alkyl substituents and the intensities vary substantially among the isomers. In addition, compounds of the same nominal mass, such as aminoacetophenone (*m/z* 246) and C3-anilines (*m/z* 246), derivatives may elute at similar retention time in the first dimension but as in this case could be well separated in the second dimension.

**Table 3:** Tentative list of aromatic amines proposed to be present in the urine of a smoker and a non- smoker based on the fragmentation patterns and the loss of iodine fragment ion *m/z* 127

Substance	*m/z* of iodinated derivatives [M^+^]	Main fragments (*m/z*)	Isomers found in urine of a smoker	Isomers found in urine of non-smoker
Diaminobenzene	330	203, 76	2	2
C1- Diaminobenzene	344	217, 90	3	2
C2- Diaminobenzene	358	231, 104	3	2
C3- Diaminobenzene	372	357, 245	3	3
C4- Diaminobenzene	386	371, 244	1	1
C1- Triaminobenzene	456	329, 202, 75	3	1
Chlorodiaminobenzene	364	237, 110	2	1
C1- Chlorodiaminobenzene	378	343, 251, 124	2	0
Bromodiaminobenzene	408	281, 154, 75	1	1
Tetrahydronaphthylamine	258	131, 102	10	2
C1- Tetrahydronaphthylamine	272	257, 130	7	7
C2- Tetrahydronaphthylamine	286	271, 159	7	10
C3- Tetrahydronaphthylamine	300	258, 173, 157	5	5
Aminoindane	244	117	5	4
C1- Aminoindane	258	244, 131	1	2
Aminoquinoline	255	128	2	2
C1- Aminoquinoline	269	142	4	3
C2- Aminoquinoline	283	156, 141	3	0
Aminopyridine	205	78	2	0
Aminothiophene	210	83	1	1
C1- Aminothiophene	224	97, 81	1	2
Aminoacetophenone	246	231, 203, 76	3	3
Aminophenol	220	93	2	1
C1- Aminophenol	234	107, 89	3	2
C2- Aminophenol	248	220, 93	1	1
C3- Aminophenol	262	247, 135	0	3
Aniline	204	77	1	1
C1- Aniline	218	91	2	2
C2- Aniline	232	217, 105	7	5
C3- Aniline	246	119	12	10
C4- Aniline	260	245, 118	17	16
Chloroaniline	238	111	2	3
C1- Chloroaniline	252	125	2	2
C2- Chloroaniline	266	231, 139	6	4
C3- Chloroaniline	280	265, 138	5	3
C4- Chloroaniline	294	279, 152	2	1
Dichloroaniline	272	145	2	2
Bromoaniline	282	155	3	2
Methoxyaniline	234	219, 92	1	1
C3- Methoxyaniline	276	261, 134	2	2
Chloromethoxyaniline	268	253, 126	1	2
Thioaniline	236	109	1	3
C1- Thioaniline	250	123, 108	1	1
Naphthylamine	254	127	2	2
C1- Naphthylamine	268	141	4	4
C2- Naphthylamine	282	155	2	2
C1- Aminobiphenyl	294	279, 152	1	1
C2- Aminobiphenyl	308	181, 166	3	4
Diaminobiphenyl	406	279, 152	1	0
Methylenedianiline	420	293, 166	1	0
C1- Diaminonaphthalene	394	267, 140	3	1

Here, the aminoacetophenone derivative (viz. iodoacetophenone, *m/z* 246) loses at first a methyl group (fragment ion *m/z* 231) followed by a carbonyl group (fragment ion *m/z* 203) and an iodo group (fragment ion *m/z* 76). In the case of the C3-aniline derivative (*m/z* 246) an iodo group is lost (fragment ion *m/z* 119) followed probably by an ethyl group (fragment ion *m/z* 91) and a methyl group (fragment ion *m/z* 77). Separation of compounds with the same nominal mass such as these is not possible when measuring with a conventional GC-MS with unit mass resolution; hence, this result underlines the importance of GCxGC-MS for the separation of multiple compound mixtures in complex matrices. Due to the large number of compounds and the GCxGC data files, which have much bigger size compared with conventional GC, analysis and handling of data, especially for quantitative analysis, is still a great challenge. In that regard, further progress has been described by Tranchida et al. [18]. We, therefore, suggest the use of a fingerprint analysis to easily monitor the exposure to aromatic amines providing a better understanding about their role on the formation of bladder cancer.

## Conclusions

An analytical method was developed to reveal a more complete picture of the occurrence of aromatic amines in urine by investigation of smoking and non-smoking persons. This method was at first developed for the detection of aromatic amines and not for quantification. More aromatic amines based on peak intensities at higher concentration were identified in the urine sample of a smoker compared with the urine of a non-smoker. Since many more aromatic amines occur in urine of a person exposed to cigarette smoke than hitherto known, it seems that the health risk potential by these compounds might exceed by far previous expectations. The use of GCxGC-MS to detect aromatic amines in urine samples was important for the identification of many structurally related compounds. The method is very efficient with high sensitivity such that single ion monitoring is not required, allowing recording of full-scan mass spectra for compound identification. In the future, quantification of aromatic amines in urine based on the presented method will be evaluated as well as the analysis of these compounds in the urine of patients who are diagnosed with bladder cancer. This eventually may be beneficial for further investigations of biomarkers, which could be used in the future to diagnose the level of cancer-causing aromatic amines in urine samples.
